# Endodontic emergencies: Your medication may be the cause

**DOI:** 10.4103/0972-0707.55623

**Published:** 2009

**Authors:** Promila Verma, Anil Chandra, Rakesh Yadav

**Affiliations:** Department of Operative Dentistry, Faculty of Dental Sciences, Chatrapati Sahuji Maharaj Medical University, Lucknow, India

**Keywords:** Formocresol, necrosis, paraformaldehyde containing paste

## Abstract

An endodontic clinician may face unwanted situations during root canal treatment. We present here an unusual case of soft tissue and gingival necrosis of the oral cavity following the use of formocresol^®^ during endodontic treatment.

## INTRODUCTION

Formaldehyde-containing medications have been used for root canal treatment for many years.[9] Various compounds containing arsenic and paraformaldehyde were used in the past when effective anesthesia could not be obtained.[11] Such agents have some clinical benefit, although local soft and hard tissue necrosis occurs if they are not confined to the pulp. The following case report describes tissue degeneration and swelling in a patient treated with formocresol during root canal treatment.

## CASE REPORT

**Figure 1:**
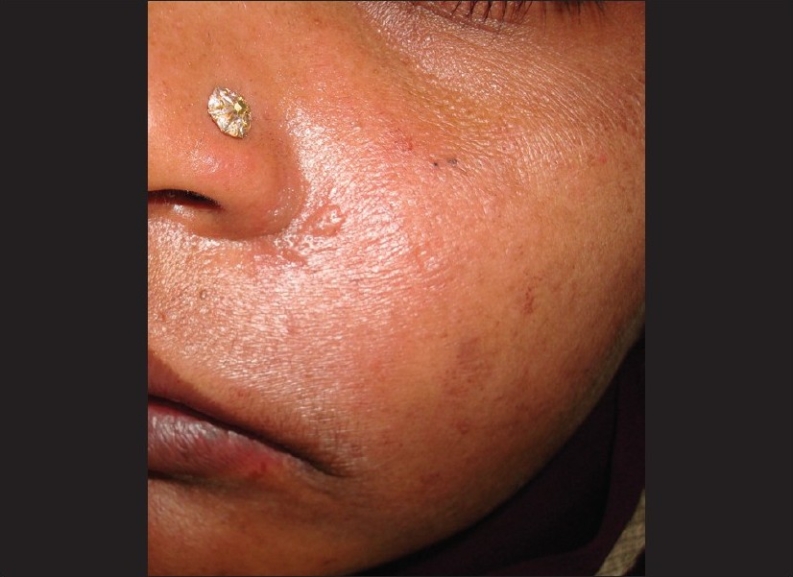
Extraoral view following the use of formocresol

**Figure 2:**
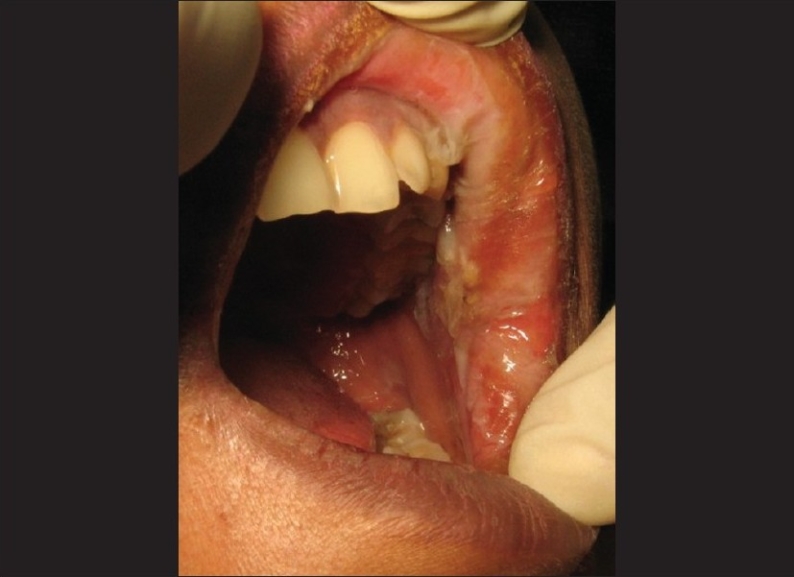
Intraoral view showing soft tissue necrosis following the use of formocresol

A 37 year-old female patient reported to the Department of Operative Dentistry in CSM Medical University with the chief complaint of pain in her maxillary left first premolar. She was diagnosed with acute irreversible pulpitis and an undergraduate student was assigned to perform her root canal treatment. She performed access opening and bio-mechanical preparation in the particular tooth and gave her dressing using formocresol-soaked cotton. The patient reported after 24 hours with the complaints of pain and swelling in her left buccal and infraorbital regions [Figure 1]. Oral examination revealed desquamation of the buccal mucosa and gingival epithelium in relation to her maxillary posterior region [Figure 2]. Extra oral examination revealed swelling in her buccal, mandibular, and infraorbital regions on the left side. There was ulceration in the angle of her mouth on the left side and her mouth opening was reduced.

### Treatment given

The first aim of treatment was to alleviate the symptoms of pain and to prevent further progress of infection. The patient was immediately advised to rinse her mouth with Betadine gargle^®^. A mixture of a steroid-based cream and Hexigel^®^ was applied all over the ulcerated surface. An analgesic was also given to relieve the symptoms of pain. The patient was prescribed antihistamines and multivitamins, which she was advised to continue for one week. She was kept on a soft diet and advised to avoid spicy food; she was recalled after one week.

**Figure 3:**
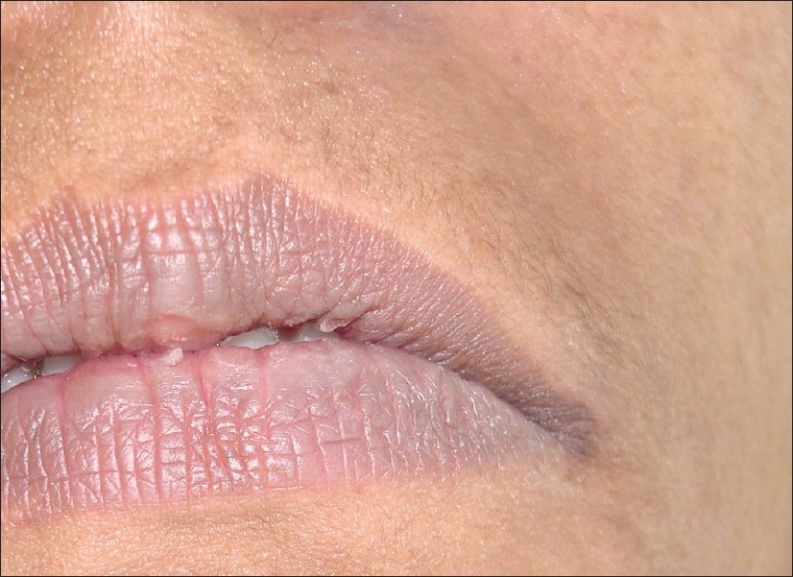
Extra-oral view after 7 days of treatment

**Figure 4:**
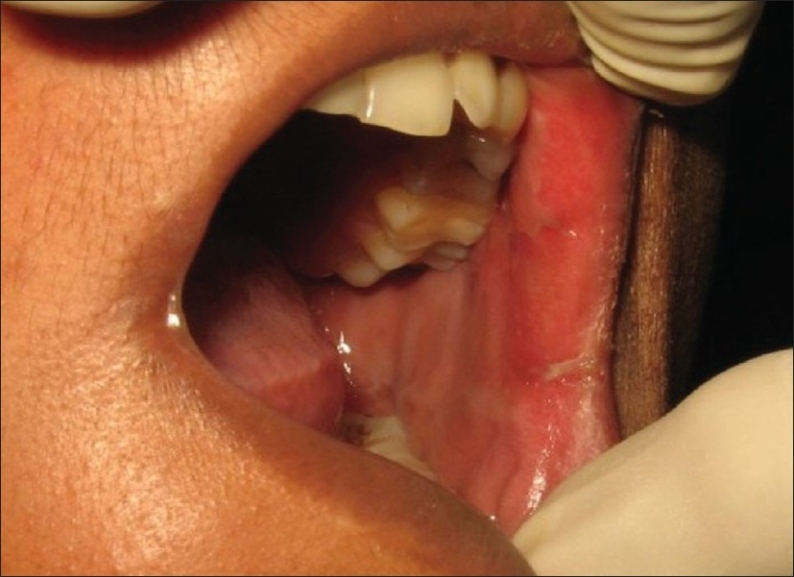
Intra-oral view after 7 days of treatment

On her subsequent visit, her condition was found to have visibly improved [Figure 3 and 4].The swelling, redness, and exfoliation of the mucosa had reduced. After the cessation of symptoms, the patient's root canal dressing was changed. By her third visit, the condition had totally resolved and her root canal treatment was subsequently completed; the tooth was permanently restored with silver amalgam.

## DISCUSSION

Formocresol was first used as a root canal medication by Buckley in 1904. It is widely used in dentistry because of its antibacterial properties in root canal disinfection.[12] It contains formaldehyde, an effective alkylating agent, and cresol, a protein-coagulating phenolic compound.[2] Its action is believed to be due to the release of formaldehyde vapors which act as a germicidal agent. Besides strong chemical disinfectant properties, cytotoxic effects have also been documented. The use of formocresol in dentistry has become a controversial issue due to its widespread distribution in the body following systemic injection,[7] and the demonstration of immune response to formocresol-fixed autologous tissue implanted in connective tissue or injected into root canals.[4,5]. Powell * et al* .[3] have shown that when formocresol was implanted subcutaneously in the connective tissue of rats, the surrounding tissue was severely damaged; causing necrosis and abscess formation. Allergies have also been reported after the application of formocresol. Formaldehyde is one of the components of formocresol that interacts with cellular proteins. The addition of cresol to formaldehyde appears to potentiate the effect of formaldehyde on protein.[6] In a study using human pulp fibroblast cultures, formaldehyde was shown to be the major component of formocresol that caused cytotoxicity and that was more toxic than cresol.[10] In this case, formocresol-soaked cotton was inserted into the pulp chamber. The resulting necrosis may have been due to excess formocresol in the cotton, which must have leaked and permeated into the surrounding tissue.[1] Betadine gargle^®^ used in this study contains Povidine iodine, which is an antiseptic. It is a complex of iodine, which kills microorganisms such as bacteria, fungi, viruses, protozoa, and bacterial spores. Povidine iodine exerts its antiseptic effect by slowly releasing iodine. Povidine iodine gargle and mouthwashes are used to treat infections of the mouth as well as throat and mouth ulcers. Topical antihistamines and corticosteroid applications meant to soothe painful ulcers may be helpful; avoiding spicy or hot foods may reduce the pain.

## CONCLUSION

Nowadays, many improved medications and anesthetics are available which obviate the need for the use of formocresol as a root canal medication or as a pulp devitalizer. Due to the caustic nature of the material, use of formocresol should be avoided.
